# Dysbiosis, inflammation, and response to treatment: a longitudinal study of pediatric subjects with newly diagnosed inflammatory bowel disease

**DOI:** 10.1186/s13073-016-0331-y

**Published:** 2016-07-13

**Authors:** Kelly A. Shaw, Madeline Bertha, Tatyana Hofmekler, Pankaj Chopra, Tommi Vatanen, Abhiram Srivatsa, Jarod Prince, Archana Kumar, Cary Sauer, Michael E. Zwick, Glen A. Satten, Aleksandar D. Kostic, Jennifer G. Mulle, Ramnik J. Xavier, Subra Kugathasan

**Affiliations:** Graduate Program in Genetics and Molecular Biology, Graduate Division of Biological and Biomedical Sciences, Emory University, Atlanta, GA 30322 USA; Department of Pediatrics, Emory University School of Medicine & Children’s Healthcare of Atlanta, 2015 Uppergate Drive, room 248, Atlanta, GA 30322 USA; Broad Institute of MIT and Harvard, Cambridge, MA 02142 USA; Department of Computer Science, Aalto University School of Science, 02150 Espoo, Finland; Department of Human Genetics, Emory University, Atlanta, GA 30322 USA; Centers for Disease Control and Prevention, Atlanta, GA 30341 USA; Center for Computational and Integrative Biology, Massachusetts General Hospital and Harvard Medical School, Boston, MA 02114 USA; Department of Epidemiology, Rollins School of Public Health, Emory University, Atlanta, GA 30322 USA

**Keywords:** Inflammatory bowel disease, Microbiome, Crohn’s disease, Dysbiosis

## Abstract

**Background:**

Gut microbiome dysbiosis has been demonstrated in subjects with newly diagnosed and chronic inflammatory bowel disease (IBD). In this study we sought to explore longitudinal changes in dysbiosis and ascertain associations between dysbiosis and markers of disease activity and treatment outcome.

**Methods:**

We performed a prospective cohort study of 19 treatment-naïve pediatric IBD subjects and 10 healthy controls, measuring fecal calprotectin and assessing the gut microbiome via repeated stool samples. Associations between clinical characteristics and the microbiome were tested using generalized estimating equations. Random forest classification was used to predict ultimate treatment response (presence of mucosal healing at follow-up colonoscopy) or non-response using patients’ pretreatment samples.

**Results:**

Patients with Crohn’s disease had increased markers of inflammation and dysbiosis compared to controls. Patients with ulcerative colitis had even higher inflammation and dysbiosis compared to those with Crohn’s disease. For all cases, the gut microbial dysbiosis index associated significantly with clinical and biological measures of disease severity, but did not associate with treatment response. We found differences in specific gut microbiome genera between cases/controls and responders/non-responders including *Akkermansia, Coprococcus, Fusobacterium*, *Veillonella*, *Faecalibacterium*, and *Adlercreutzia*. Using pretreatment microbiome data in a weighted random forest classifier, we were able to obtain 76.5 % accuracy for prediction of responder status.

**Conclusions:**

Patient dysbiosis improved over time but persisted even among those who responded to treatment and achieved mucosal healing. Although dysbiosis index was not significantly different between responders and non-responders, we found specific genus-level differences. We found that pretreatment microbiome signatures are a promising avenue for prediction of remission and response to treatment.

**Electronic supplementary material:**

The online version of this article (doi:10.1186/s13073-016-0331-y) contains supplementary material, which is available to authorized users.

## Background

Inflammatory bowel disease (IBD), including Crohn’s disease (CD) and ulcerative colitis (UC), is characterized by chronic remitting and relapsing inflammation of the gastrointestinal tract. Persistent inflammation and continuing insult lead to fibrosis, scarring, and the need for multiple surgeries. The pathogenesis of IBD is complex and poorly understood. A disturbance of intestinal mucosal homeostasis, influenced by genetic factors, the intestinal microbiome, the immune system, and environmental exposures, is believed to underlie IBD [1, 2]. While 200 distinct genetic loci have been associated with IBD in a recent report [3], many of these genes point to pathways involving bacterial recognition or host response to microbial infections, both clearly influenced by the environment. Although the prevalence of adult-onset IBD has plateaued in the Westernized world, recent population-based studies on IBD from Canada [4], the USA [5], and Europe [6] suggest a rapid increase in pediatric-onset IBD, particularly in children younger than 10 years. Genetic causes are unlikely to account for these epidemiological findings. The risk of IBD among first-generation immigrants to the Western world from south Asia and Africa as well as the prevalence of IBD in native Asia or Africa are exceedingly low, yet second-generation immigrants have a greatly increased risk similar to that in the location to which they immigrated [7]. This emerging global rise of pediatric IBD incidence has fueled a quest to identify early life exposures including potential microbiome alterations due to lifestyle and diet that could explain the increasing risk for IBD among children [8, 9].

Several studies have described characteristic patterns within the gut microbiome of patients with IBD [10–13]. In general, shifts in bacterial taxa and decreased community diversity have been found in treatment-naïve CD [14] and in IBD in general [15–17], with the extent of dysbiosis associated with severity of inflammation [18]; however, it is not clear whether these changes are a cause or consequence of IBD [2]. In one recent study involving a large number of subjects, the microbiome of treatment-naïve pediatric CD patients had a distinct signature compared to non-IBD subjects, as measured by both fecal and intestinal mucosa bacterial ecosystems [19]. However, this study used primarily mucosal biopsies and was limited to a single time point—it did not capture the dynamics of the gut microbiome over time. One recent study showed that dysbiosis results from independent effects of inflammation, diet, and antibiotics after selected subjects with pediatric Crohn’s disease were treated with enteral nutrition and some conventional medications [18]. Although this study measured the bacterial community before and after intervention, the study only provided data for an 8-week study period and only 4 samples per patient. Long-term data are still lacking regarding dysbiosis subjects who undergo standard-of-care treatment in clinical practice. Once IBD is diagnosed, patients undergo a series of treatments to induce clinical remission, in which mucosal healing is promoted by controlling mucosal inflammation. Some patients respond clinically to treatment with normalization of symptoms and evidence of mucosal healing seen in repeat colonoscopies (“responders” or “remitters”); other patients continue to have persistent inflammation or a remitting-relapsing disease course with a variable degree of mucosal inflammation (“non-responders” or “non-remitters”). It is critically important to study the intestinal microbiome over the course of treatment to identify whether there are microbial signatures that distinguish these different outcomes. This can be achieved with longitudinal microbiome analysis, starting at diagnosis and following up throughout treatment in parallel with clinical characterization. We hypothesize that distinct signatures of microbiota can be found and applied in clinical practice to assess ongoing inflammation and predict response to treatment. An important study by Kolho et al. examined the treatment responses using fecal calprotectin in patients with median disease duration of 3.5 years after diagnosis [20]. Although our study was similar, our study design differed from that of Kolho et al. in that we used mucosal healing in addition to fecal calprotectin as a measure of mucosal inflammation and used sequencing rather than phylogenetic microarrays to classify species levels.

Here we report the results of a longitudinal investigation of 19 children diagnosed with IBD, of whom 15 had a final diagnosis of CD and 4 had a final diagnosis of UC. All 19 subjects were recruited from a single center, were treatment-naïve at the time of enrollment, were treated with current standards of practice guidelines, and were followed clinically for a median of 8 months. Treatment regimens were not protocolized, but treatment was escalated to maximal medical therapy or surgical resection was recommended if, upon clinical evaluation, the subject was categorized as a non-responder to previous treatment. We also recruited and followed 10 unaffected controls for comparison: 6 family members and 4 unrelated controls. We measured fecal calprotectin in all samples as an objective measure of inflammation as well as the subjective clinical disease activity indices (Pediatric Crohn’s Disease Activity Index [PCDAI] or Pediatric Ulcerative Colitis Activity Index [PUCAI]). The strength of our study lies in the dense longitudinal data collection (217 total visits—a median of 8 time points for both cases and controls), thorough clinical characterization of our patients at each visit, measurement of clinical disease activity indices, and simultaneous use of fecal calprotectin as an objective measure of mucosal inflammation. We comprehensively analyzed inflammation, diversity, and dysbiosis by standard methods including the previously described dysbiosis index, explored gut microbiome differences at the genus level among cases and controls and treatment responders and non-responders, and finally assessed the ability of pretreatment samples to predict treatment response.

## Methods

### Study population

Potential participants were identified from Children’s Healthcare of Atlanta inpatient wards and outpatient pediatric IBD clinics based on clinical suspicion of IBD based on symptoms or lab work. Criteria to participate in the study included CD or UC diagnosis confirmed by colonoscopy and/or magnetic resonance enterography, willingness to participate, and ability to maintain close follow-up. Patients and families gave informed consent and assent to participate in the study. Exclusion criteria included prior diagnosis of IBD, prior therapy with immunomodulators or biologics, or history of non-compliance with clinical appointments.

A total of 19 pediatric IBD cases (≤17 years old, 15 with CD and 4 with UC) were enrolled in this longitudinal prospective study between June 2013 and January 2014. Participants were followed at regular intervals beginning at the time of enrollment until the termination of the study in August 2014. All patients were phenotyped at the time of enrollment according to the Paris Classification [21]. Demographic and phenotypic characteristics were collected via patient interview and chart review at the time of sample delivery, and an abbreviated PCDAI [22–24] or PUCAI was obtained at all clinical visits [25]. Medical treatment was not affected by joining this study. Patients started to receive treatment between their first and second clinical visits. Patients were treated with aggressive monotherapy of either immunomodulators or biologics with mucosal reassessment via colonoscopy approximately one year after diagnosis. Based on the presence or absence of mucosal healing, we dichotomized patients as responders (*n* = 6) or non-responders (*n* = 13), respectively, independent of any knowledge about microbiome composition. Since subjects received multiple treatments, we did not categorize based on the particular treatment exposures. Patients receiving surgery were classified as non-responders, and only presurgery time points were used in analyses. Family members of patients were recruited as related controls (*n* = 6), and unrelated controls ≤17 years old with no IBD diagnosis were also recruited (*n* = 4). Once enrolled, participants were followed no more frequently then weekly.

### Specimen collection and processing

**Fig. 1:**
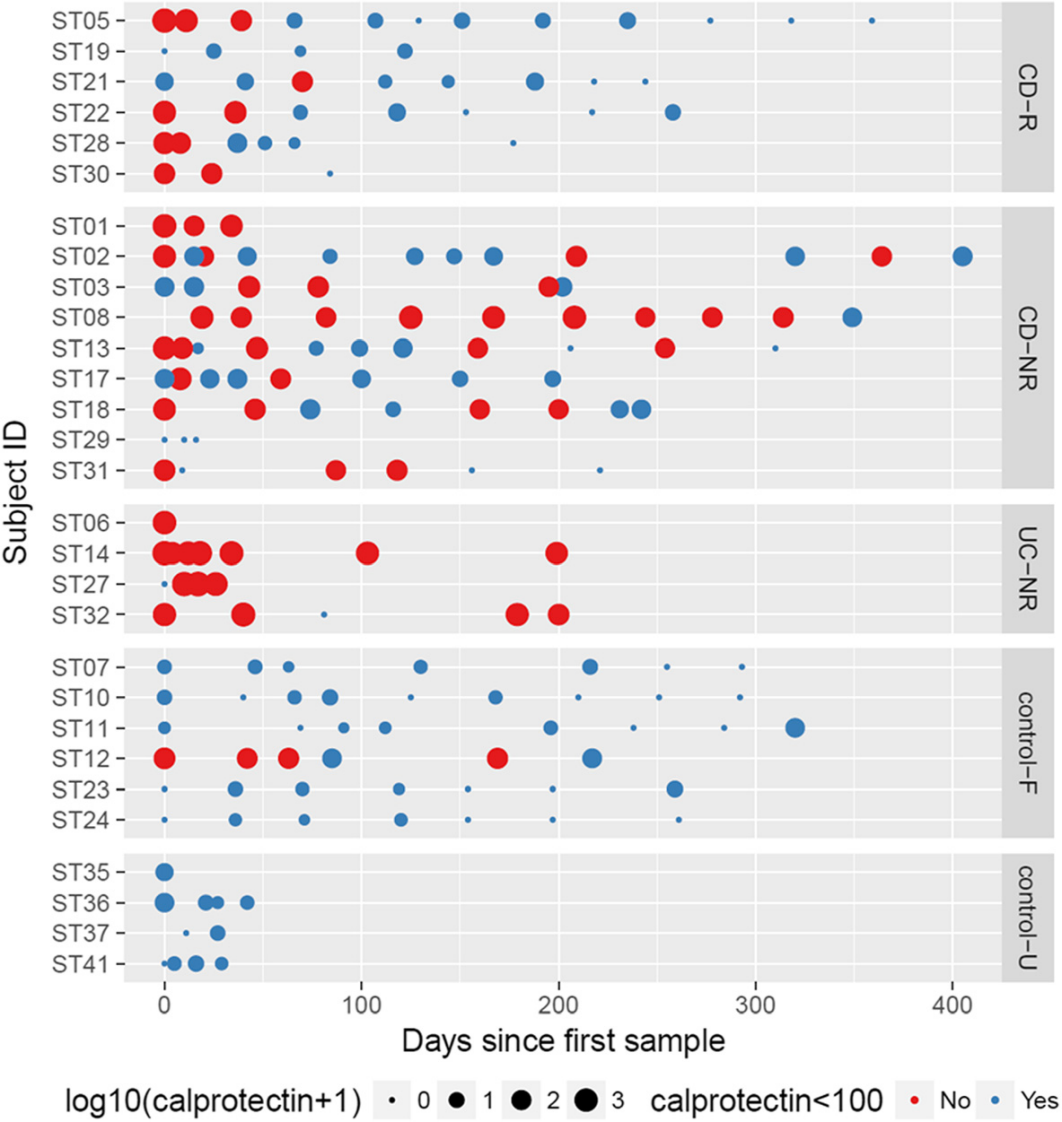
Log_10_(calprotectin + 1) values for all study subjects used in analysis. Larger circle size reflects higher measured calprotectin. Time points where calprotectin was <100 μg/g are shown in *blue*; time points where calprotectin was >100 μg/g are shown in *red. CD* Crohn’s disease, *UC* ulcerative colitis, *R* responder to treatment, *NR* non-responder to treatment, *F*, familial control, *U* unrelated control. (See also Table 1 and Additional file 2: Table S1.)

Fecal samples were obtained at regular intervals beginning at the time of diagnosis and throughout the study (Fig. 1). Each fecal sample was collected and placed into two separate Para-Pak Vials: one with 100 % ethanol and one without ethanol. The specimen with ethanol was submitted to the study coordinator at room temperature for processing within 24 hours of collection. The specimen was spun down, the ethanol discarded, and the remaining stool was either stored at –20 **°**C until ready for aliquoting or immediately aliquoted to be stored at –80 **°**C for fecal microbiome analysis. The specimen without ethanol was stored at –20 **°**C until it was aliquoted and stored at –80 **°**C for fecal calprotectin analysis. Fecal calprotectin was measured by Eagle Biosciences Calprotectin Enzyme-Linked Immunosorbent Assay (ELISA) kits according to the manufacturer’s guidelines.

### Bioinformatic processing

In collaboration with the Broad’s Molecular Biology R&D (MBRD) lab, we sequenced the V4 region of the bacterial 16S rRNA gene using the Illumina MiSeq platform according to the manufacturer’s specifications. Reads were demultiplexed into fastq files for each sample using sequence barcodes. Forward and reverse reads were joined with PANDAseq [26]. After samples with fewer than 3000 reads were excluded, there was a median of 66,000 reads per sample used in the study. The joined sequence files were formatted using a Python script to add QIIME headers with the respective sample ID to each sequence before concatenating into one file for input into QIIME 1.8.0 [27]. Operational taxonomic units (OTUs) were picked using the QIIME pick_closed_reference_otus.py script with a threshold of 97 % identity to the Greengenes v13_8 database. A median of 91 % of reads per sample were classified successfully with this closed-reference OTU approach. The Shannon alpha diversity was calculated on the unfiltered biom table using the alpha_diversity.py script, and weighted UniFrac distances were calculated with the beta_diversity.py script. The microbial dysbiosis index (initially described by Gevers et al. [19]) was calculated in R for each sample. The microbial dysbiosis index is defined as the log_10_ of the total abundance in organisms increased in CD divided by the total abundance of organisms decreased in CD. The increased-in-CD taxa comprise *Enterobacteriaceae*, *Pasteurellaceae*, *Fusobacteriaceae*, *Neisseriaceae*, *Veillonellaceae*, and *Gemellaceae*. Decreased-in-CD taxa are *Bacteroidales*, *Clostridiales* (excluding *Veillonellaceae*), *Erysipelotrichaceae*, and *Bifidobacteriaceae* [19].

To test the robustness of our findings from these Shannon diversity and dysbiosis calculations, we repeated association tests between cases and controls using our data (1) with a de novo OTU clustering approach and (2) by rarefying to an even sequencing depth. Our de novo analysis was performed the same as our original closed-reference analysis with the exception that chimeras were first removed from each sample using USEARCH v6.1 [28], then OTUs were picked using the pick_de_novo_otus.py script. Taxonomic classification was performed using the same Greengenes database. The same median percentage of sequences was ultimately successfully classified (91 %) using this de novo approach.

We randomly rarefied each sample in our original closed-OTU biom table to 3155 sequences, the lowest sequencing depth observed in our samples, using the rrarefy function in the R package vegan [29]. We then measured the Shannon diversity using vegan’s diversity function and calculated the dysbiosis index using the same R code described previously. We repeated this 10,000 times and took the median of the results from these rarefactions for each sample; we then repeated our regression analyses using these values. For a complete summary of reads/sample, QC information, and calculated values, see Additional file 1.

Overall there were 7628 OTUs in our samples. For our genus-by-genus and random forest analyses we collapsed data to the genus level (combining OTUs belonging to the same genus) and converted counts to frequencies using the summarize_taxa.py QIIME script. There were 397 genus-level taxa in our 158 microbiome samples. To test for significance, we required a genus to be present at greater than 0.15 % abundance in at least one sample, leaving 134 genera.

### Statistical analysis

We performed all data analyses in R. To account for the correlations within individuals over time, we performed linear regressions in a generalized estimating equation (GEE) framework [30] using the R package geepack [31]. We assumed an independent correlation structure and used the robust (sandwich) estimator for standard error. Subject observations were additionally inversely weighted by the total number of observations for that individual to ensure that results were not driven by individuals who were observed more frequently [32]. Wald tests were used to assess the significance of coefficients in our GEE. To compare marker levels between groups, we modeled markers (calprotectin, dysbiosis, diversity) as a function of disease status (case versus control or UC versus CD). To assess differences between groups at baseline (all clinical outcomes as well as genus-by-genus analysis), or to measure changes over time, we considered models with time since study enrollment. When comparing change over time between CD, UC, and controls, time by diagnosis interactions were also considered. We used the same models without time to assess average differences between groups over the course of disease. For associations between pairs of markers (e.g., calprotectin and dysbiosis) throughout the course of our study, we modeled one marker (calprotectin) as a function of the other marker (dysbiosis).

### Predictive modeling

We used the R package randomForest [33] and genus frequency data from each subject’s first pretreatment fecal sample (available for 5 responders and 12 non-responders) to train a random forest with 25,001 trees to predict response or non-response. Trees were grown to the maximum size possible; by default, 12 genera (the square root of the number of input genera) were considered as candidates at each split, and splitter importance was calculated as mean decrease in the Gini impurity, described in the randomForest documentation [33]. Because of the small sample size, we did not differentiate between UC and CD patients for this analysis. To assess if this was reasonable, we calculated the proportion of the variance in weighted UniFrac distances between patients’ pretreatment samples explained by response/non-response status and IBD subtype using permutational ANOVA (PERMANOVA) as implemented in the adonis function in the R package vegan [29]. To account for unequal sample sizes of responders and non-responders in our random forest, we used weights equal to the inverse of the sample size of each class; the cost of misclassifying responders therefore equaled the cost of misclassifying non-responders. We also performed the analysis with equal class sizes (5 each of responders and non-responders) to ensure that our results were not the result of the class imbalance of our cohort. The receiver operating characteristic (ROC) curves and the area under the ROC curves (AUC) were generated using the ROCR package in R [34]. The significance of prediction accuracy and AUC was assessed by permuting the response/non-response status 10,000 times.

## Results

### Extensive characterization of gut inflammation and microbiome in a longitudinal cohort of children with IBD

Twenty-nine individuals were included in the longitudinal analysis, representing four groups: patients with CD (*n* = 15), patients with UC (*n* = 4), unaffected controls with a first-degree genetic relationship to an affected individual (family members, *n* = 6), and unaffected controls with no genetic relationship to any affected individual included in this study (unrelated, *n* = 4). Table 1 shows a summary of clinical characteristics and total number of visits used in the analyses for all study participants. A more detailed summary of number of microbiome measures, calprotectin values, and PCDAI time points by case/control group is provided in Additional file 2: Table S1. Figure 1 shows a comprehensive visualization of calprotectin measures for all patient and control time points used in all analyses. GEE comparison of familial and unrelated controls showed no significant differences at baseline and no differences in average fecal calprotectin or alpha diversity between the two groups. However, on average unrelated controls had a higher dysbiosis index than related controls (Additional file 2: Table S2). These groups were pooled into one group of controls for all subsequent analyses, so our results were not inflated by the lower dysbiosis index apparent in related controls.

**Table 1:** A summary of relevant characteristics for study participants

Cases			
Diagnosis	Crohn's disease	15 (78.9 %)	Count (%)
	Ulcerative colitis	4 (21.1 %)	
Treatment outcome	Response/mucosal healing	6 (31.6 %)	
	Non-response without surgery	8 (42.1 %)	
	Non-response with surgery	5 (26.3 %)	
Time points	Microbiome	6 (1–12)	Median (range)
	Calprotectin	6 (1–12)	
	PCDAI	7 (3–13)	
Controls			
Relatedness	Familial	6 (60 %)	Count (%)
	Unrelated	4 (40 %)	
Time points	Microbiome	5 (1-8)	Median (range)
	Calprotectin	6.5 (1–9)	
	PCDAI	NA	

### Subjects with IBD have increased markers of inflammation and dysbiosis compared to controls

**Fig. 2:**
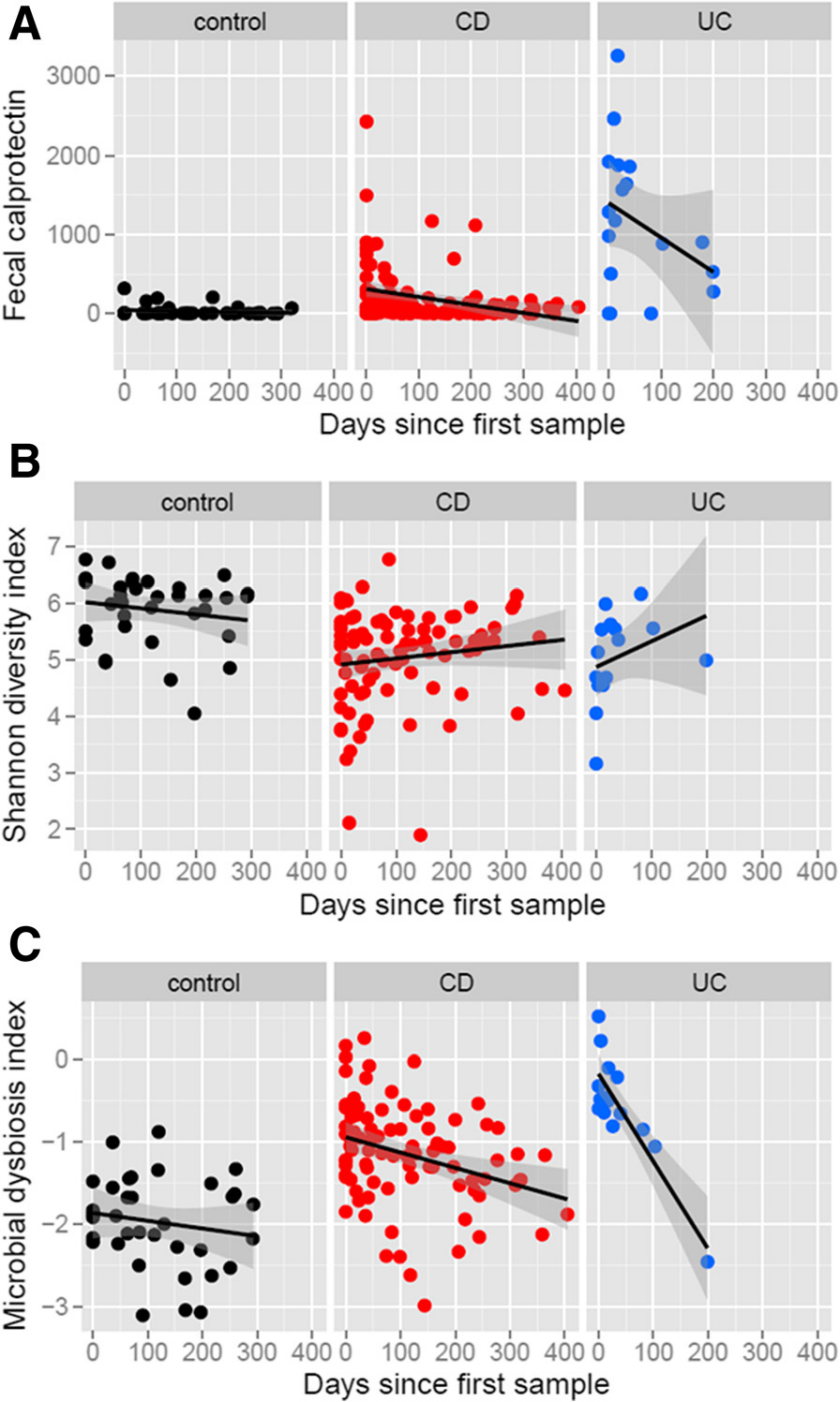
Clinical characteristics for all study subjects. **a**–**c** Characteristics for control subjects (*black*), Crohn’s disease patients (*CD, red*), and ulcerative colitis patients (*UC, blue*) are plotted over time with unadjusted regression lines in *black* and 95 % confidence intervals in *gray*. For patients with CD and UC, calprotectin decreases (**a**), alpha diversity increases (**b**), and gut microbial dysbiosis decreases (**c**) over time, reflecting overall improvement following treatment. Additionally, calprotectin and microbial dysbiosis were significantly higher in our UC patients than in CD. (See also Additional file 2: Figures S1 and S2, Tables S3 and S4.)

We first tested general differences in inflammation, microbiome diversity, and microbial dysbiosis between IBD cases and controls using our weighted GEE approach to properly control for correlations within individuals. The significance of these coefficients was assessed via Wald tests. Additional file 2: Table S3 summarizes beta and *p* value information for comparisons of baseline values (including time since first sample as a covariate) and overall averages. Figure 2 shows calprotectin, alpha diversity, and dysbiosis for all time points for controls, CD patients, and UC patients (Additional file 2: Figure S1 shows all time points summarized in box-and-whisker plots; Additional file 2: Figure S2 shows controls, responders, and non-responders over time with a different color for each individual).

For controls, baseline calprotectin was 42 ± 99 μg/g. Patients with CD had fecal calprotectin values 313 μg/g higher at baseline than controls (*p* = 0.0002), and patients with UC had values 1330 μg/g higher than controls (*p* = 4E-11; Additional file 2: Table S3 summarizes all CD/UC/control comparisons). Over the entire course of our study the average difference in fecal calprotectin for CD and UC patients compared to controls was 181 μg/g (*p* = 0.00002) and 1100 μg/g (*p* = 4E-10), respectively. As seen in previous studies, patients with IBD had overall lower alpha diversity as measured by the Shannon index. The Shannon index at baseline for controls was 6.02 ± 0.58. Patients with CD had Shannon index values 0.94 lower at baseline (*p* = 0.00001) and 0.72 lower on average (*p* = 0.007) relative to controls. Patients with UC had Shannon values 1.31 lower at baseline (*p* = 8E-05) and 0.98 lower on average (*p* = 0.002).

Our sample of patients with IBD also had significantly higher scores on the dysbiosis index than controls. At baseline, the mean control dysbiosis index was –1.85 ± 0.55. Baseline dysbiosis was 0.86 point higher for CD patients (*p* = 6E-8) and 1.75 points higher for those with UC (*p* = 4E-15). Dysbiosis scores were on average 0.67 point higher in CD (*p* = 3E-07) and 1.38 points higher in UC (*p* = 3E-10).

Our microbiome findings of decreased Shannon diversity and increased dysbiosis did not change when we calculated these values after de novo OTU picking or after taking the median of 10,000 rarefactions to the lowest sequencing depth seen in our closed biom table (see Additional file 3 for a comparison of these approaches to results of our original closed-reference OTU approach).

Patients with UC had significantly higher calprotectin and dysbiosis indices than those with CD (Fig. 2, Additional file 2: Table S4). UC patients also had fecal calprotectin levels 829 μg/g higher at baseline (*p* = 2E-05) and 917 μg/g higher on average (6E-06) compared to CD patients. The dysbiosis index was 0.49 point higher among UC patients at baseline (*p* = 0.02) and 0.70 point higher on average (0.0007) than in CD patients. While the Shannon diversity was lower in our patients with UC, this difference was not significant, possibly due to the relatively small sample size of our cohort.

Our longitudinal samples also show improvements in outcome measures over time for IBD patients (Fig. 2), reflecting overall response to treatment, while these measures did not significantly change for controls over the course of the study (Additional file 2: Table S3). Calprotectin declined in patients with CD relative to controls (*p* = 0.02), and in those with UC, calprotectin declined at around four times the rate of CD compared to controls (*p* = 3E-06). An increase in Shannon diversity relative to controls was not significant for CD patients, but Shannon diversity did improve over the course of the study for patients with UC compared to controls (*p* = 0.002). Both CD and UC patients showed improvements (decreases) in the microbial dysbiosis index compared to controls (*p* = 0.03 and *p* = 1E-13, respectively), with UC patients having a higher comparative rate of decline.

### Dysbiosis associates significantly with clinical and biological measures of disease severity

Our next aim was to test whether dysbiosis showed an association with calprotectin in our cohort. Using GEE, we found that higher dysbiosis associated significantly with higher calprotectin (Additional file 2: Table S5). In the overall dataset including both cases and controls, one unit increase in microbial dysbiosis (overall mean –1.3 ± 0.74) was associated with a 260-point increase in calprotectin (*p* = 0.0004). This finding also held true when examining cases only: a one-unit increase in dysbiosis (case mean –1.06 ± 0.66) associated with 286 μg/g higher calprotectin (*p* = 0.02, Additional file 2: Figure S3A). This is the first time the dysbiosis characteristic of the CD gut microbiome has been linked to a clinical measure of inflammation: fecal calprotectin. In contrast, we found that the Shannon alpha diversity did not show a relationship with calprotectin (Additional file 2: Table S5). Our results were not impacted by using a de novo OTU-picking approach or rarefying reads from each sample from the closed-OTU-picking biom file to even depth (see Additional file 3).

For our Crohn’s patients, dysbiosis also significantly associated with increased PCDAI, the current clinical measure of disease activity (*p* = 0.0001, Additional file 2: Figure S3B). However, PCDAI did not associate significantly with calprotectin (Additional file 2: Table S5, Additional file 2: Figure S3C), suggesting that PCDAI is not a good stand-in for a direct measure of inflammation such as calprotectin.

### Gut microbiome differences between groups

While the dysbiosis index has predictive power of whether an individual has CD [19], we found that the baseline dysbiosis index was not significantly different (*p* = 0.3) between treatment responders, who showed evidence of mucosal healing (*n* = 6), and non-responders (*n* = 13). This finding suggests that baseline dysbiosis may identify cases, but may not be the best tool for predicting actual response to treatment. Because the components of the dysbiosis index are broad categories (i.e., family- and order-level taxa), we next used GEE (again with Wald tests for coefficient significance) to test whether distinct microbiome signatures could be identified among responders and non-responders at the genus level. Using GEE allowed us to leverage the power of all of our time points to test differences, both between cases and controls and between non-responders and responders.

**Fig. 3:**
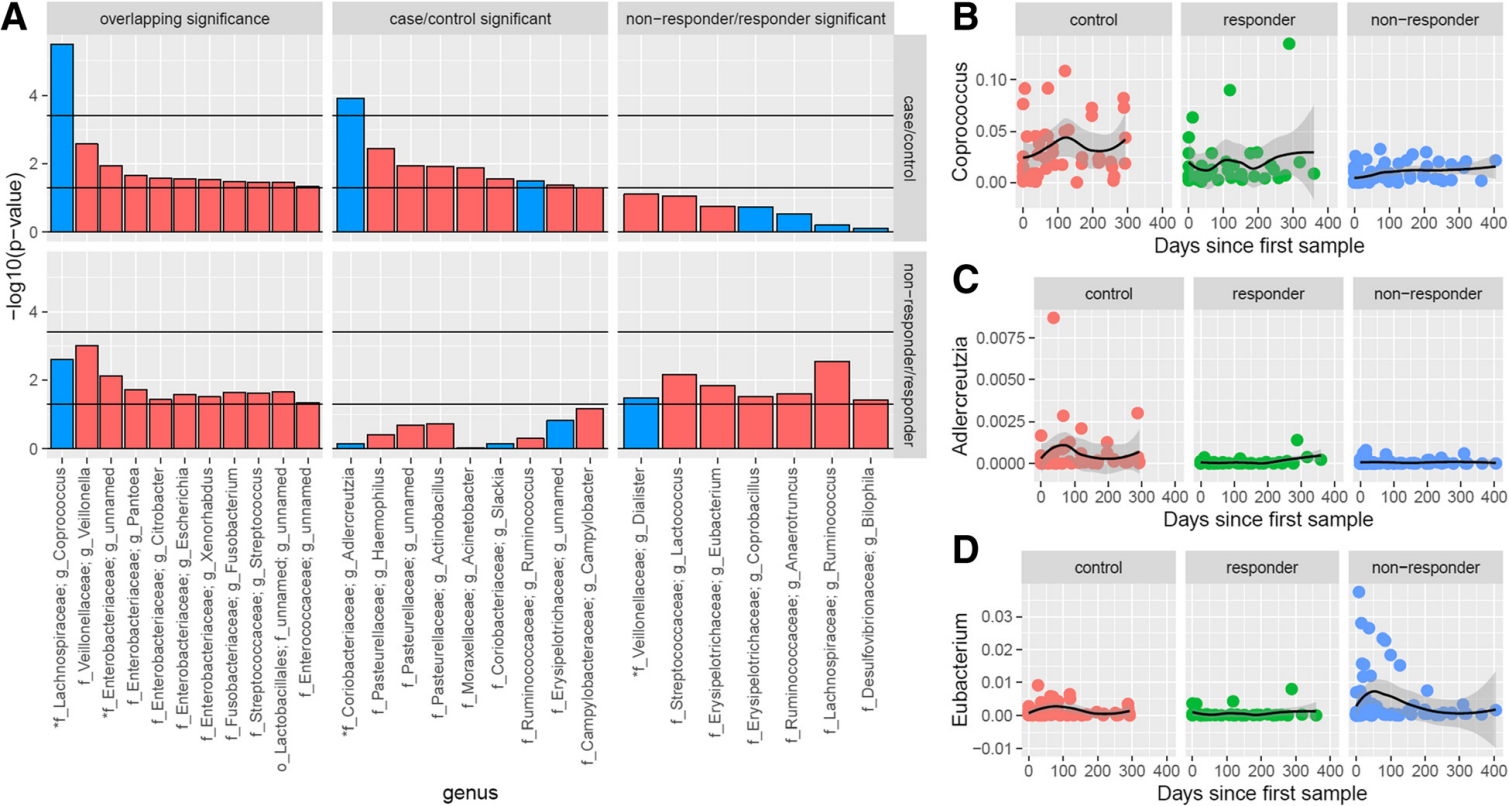
Genera with significant differences between cases and controls, non-responders and responders. **a** –Log_10_(*p* value) from testing difference in abundance of each genus in cases compared to controls and non-responders compared to responders. *Blue bars* indicate taxa negatively associated with case or non-responder status, and *red bars* indicate a positive association. The *line* below 2 represents the threshold for nominal significance; the *higher line* is the significance level after Bonferroni adjustment for multiple tests. The *asterisks* denote taxa that also appear in the results of our random forest classifier. **b**–**d** Example patterns representative of each of the three categories: **b** significant in both comparisons, **c** significant only between cases and controls, and **d** significant only between non-responders and responders. (See also Additional file 2: Table S6.)

We found that 20 genera had nominally significantly different abundance (*p* ≤ 0.05) between cases and controls at baseline. Interestingly, 7 of these 20 genera were not captured by the dysbiosis index. We also found 18 genera that differed significantly at baseline between responders and non-responders, 5 of which were not captured in the dysbiosis index. The taxa that differ between groups are summarized in Fig. 3 and Additional file 2: Table S6.

When we compared the list of significantly different genera between cases and controls to the significant genera from our non-responder/responder comparison, 11 of these taxa overlapped. The direction of effect in all overlapping taxa was the same in the two comparisons: if a genus was significantly increased in cases compared to controls, that genus was likewise increased in our non-responders compared to responders.

Because of our limited sample size, this analysis was largely exploratory: only two taxa, *Coprococcus* and *Adlercreutzia*, met the threshold for significance in the case/control comparison (no taxon met this threshold in our non-responder/responder comparisons) after conservative Bonferroni correction for multiple tests, with a significant *p* value defined as <0.05/134. *Coprococcus* was decreased in cases compared to controls and further decreased in non-responders compared to responders. *Adlercreutzia* was also decreased in cases compared to controls but was at similar levels in non-responders and responders. While the association of *Coprococcus* with IBD has long been known, the association with *Adlercreutzia* has not been previously reported.

### Predicting future response to treatment via the gut microbiome using pretreatment samples

**Fig. 4:**
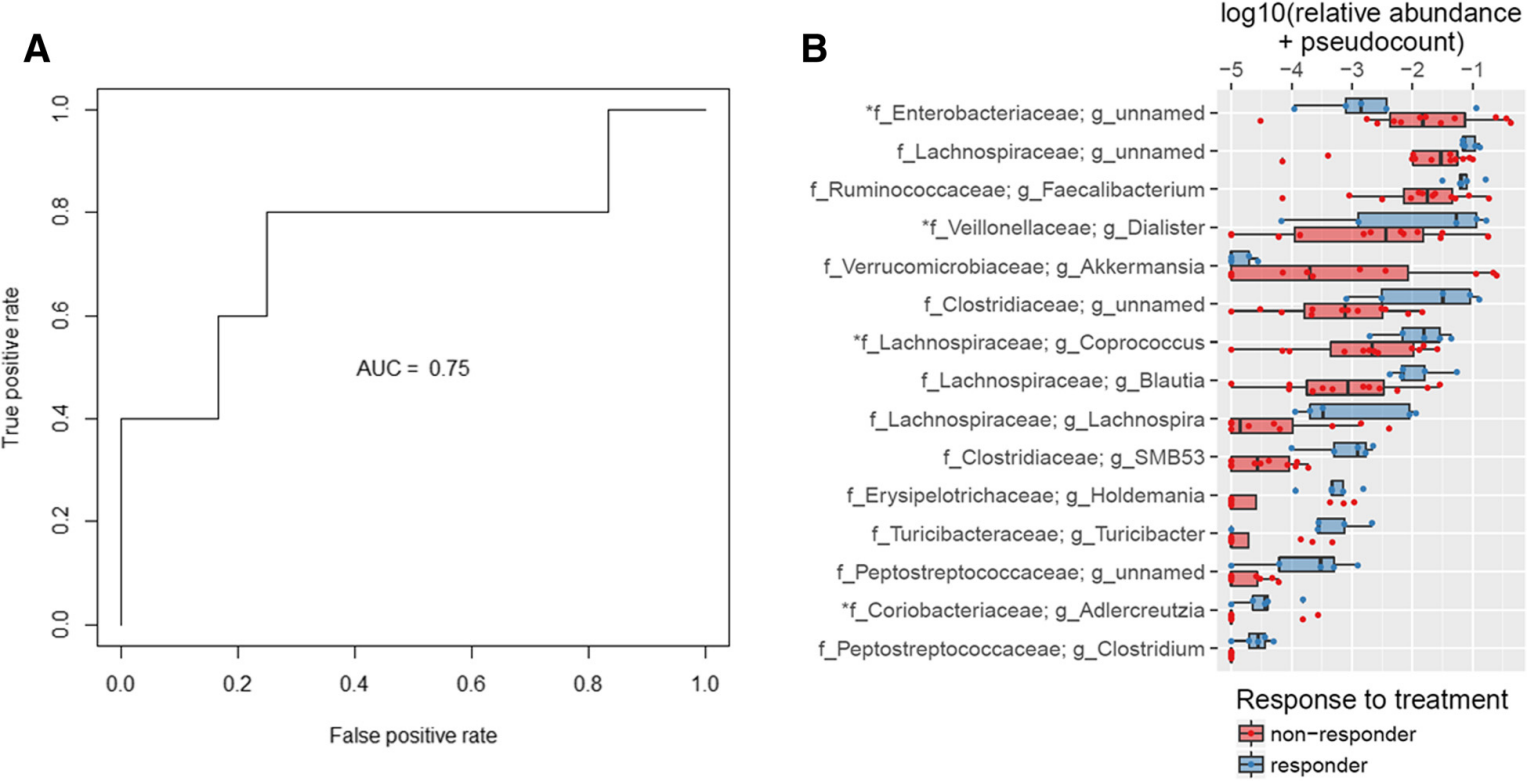
Use of genera to predict eventual response to treatment in pretreatment samples. **a** Our classifier classifies response status significantly better than random guess with AUC = 0.75 and overall accuracy of 76.5 % for predicting treatment response/non-response. **b** Box plots of the log_10_ relative abundance plus pseudocount (1E-05) of the 15 genera with highest importance scores in random forest analysis in responders and non-responders. The *asterisks* denote taxa also identified as significant in our generalized estimating equations analysis. (See also Additional file 2: Figures S4 and S6, Additional file 2: Tables S7 and S9.)

We used a random forest classifier to determine if treatment response among cases could be predicted using microbiome data from the first pretreatment sample from each individual. Five responders and 12 non-responders had pretreatment samples for analysis. We combined patients with UC and CD because the IBD subtype explained only 4 % of the variability in the weighted UniFrac distance between pretreatment samples after accounting for responder/non-responder status, which explained 23 % of the variability (*p* = 0.01 after 10,000 permutations). Our classifier attained an area under the ROC curve (AUC) of 0.75 (Fig. 4a) and 76.5 % accuracy of prediction (significant at *p* = 0.04 and *p* = 0.03, respectively, after 10,000 permutations of treatment response/non-response status). The confusion matrix and precision-recall curves for our random forest model can be found in Additional file 2: Table S7 and Additional file 2: Figure S4, respectively. Because the prediction error among responders in this model is high (60 %), we were concerned that only non-responders had a distinctive pattern; this could also lead to a higher prediction error (lower accuracy) than reported here among populations having a higher proportion of responders. To investigate this, we additionally used a subsampling approach to fit our random forest classifier, so that each tree was fit using 5 responders and 5 non-responders. This model has the same overall prediction accuracy (76.5 %), but the prediction error in responders (20 %) and non-responders (25 %) is more comparable, suggesting that both responders and non-responders have distinct OTU profiles. These results also suggest that the prediction accuracy we report here is achievable even in populations with varying proportions of responders. The confusion matrix for the subsampled model can be found in Additional file 2: Table S8; the ROC and precision-recall curves can be found in Additional file 2: Figure S5.

The abundances of genera with the top 15 highest variable importance scores in our weighted random forest (listed with importance scores in Additional file 2: Table S9) are shown in Fig. 4b. Additional file 2: Figure S6 shows stacked bar charts for each sample used in the random forest (categorized by eventual response or non-response) summarizing those of the top 15 genera that were found above 1 % average abundance. Four of the top 15 genera (*Coprococcus*, *Adlercreutzia*, *Dialister*, and an unnamed genus of *Enterobacteriaceae*) overlapped with our GEE results. This overlap is denoted with asterisks in Fig. 3a and Fig. 4b. Three of these genera were the most significant in our GEE groupings, further implicating their significance in our IBD patients: *Coprococcus* was most significant of the genera in both case/control and responder/non-responder comparisons, *Adlercreutzia* was most significant in the case/control comparisons, and *Dialister* the most significant in responder/non-responder comparisons. Furthermore, *Coprococcus* and *Adlercreutzia* were the two genera that remained significant in our case/control analysis (both with decreased abundance) after Bonferroni correction of our GEE results. Importantly, 14 of the top 15 most important genera identified are identical between the weighted and equal sampling analyses (Additional file 2: Table S10), supporting the conclusion that these taxa are truly responsible for separating responders and non-responders in our cohort. Replication in a larger study will be needed to confirm the role of these taxa in treatment response.

## Discussion

We conducted the largest longitudinal study published to date following newly diagnosed IBD subjects in real time, collecting measures of disease activity, mucosal inflammation, and microbiome composition. Sample collection was initiated at diagnosis, prior to treatment, and continued throughout the medical and surgical management of these patients. Here we show that (1) longitudinal stool sampling was both feasible and robust; (2) microbial dysbiosis improved from baseline but persisted despite complete cessation of clinical disease activity among responders; (3) distinct microbiota signatures emerged among responders compared with non-responders at the genus level, but not dysbiosis index; and (4) treatment-naïve analysis of the microbiome could potentially be used to predict whether a subject will respond to treatment. Our study was based on real day-to-day clinical practice, so the study design did not impact treatment choices for the subjects. Using this approach, our patients could be treated in a manner consistent with standard of care. Our findings may prove clinically useful in tailoring therapies; if confirmed by a larger study, clinicians could, in the future, make microbiome-informed decisions about early escalation of medical therapies versus timely surgical interventions.

In our study, we focused on following patients over time using stool samples, because obtaining repeated biopsy samples in a clinical setting is not feasible—it is invasive, expensive, and impractical for day-to-day clinical practice. We show that repeated stool samples can depict the diversity and dysbiosis of the microbiome. This is an important implication for future studies, because it suggests that stool samples, which are relatively cheap and easy to acquire, are an appropriate substitute for biopsy samples to monitor the microbiome of patients with IBD.

In terms of clinical outcomes, we assessed disease activity with PCDAI/PUCAI, the current standards in clinical use. These measures largely rely on clinician observation and patient self-report and are therefore indirect assessments of disease activity. Since inflammation impacts microbiome indices, many studies have been criticized for not having an objective measure of inflammation. To address this shortcoming, we measured fecal calprotectin as a proxy for mucosal inflammation [35, 36]. Fecal calprotectin is a quantitative measure of disease activity that is not affected by self-reporting bias and is a direct biomarker of mucosal inflammation, the trademark of IBD.

Previously, Gevers et al. [19] described the gut microbiome in treatment-naïve CD patients and created the dysbiosis index to reflect the distinct alteration of the microbiome in CD. We applied the dysbiosis index to our population and further showed it to be a useful and relevant tool: the dysbiosis index was significantly higher (indicating more dysbiosis) in both our CD and UC subjects compared to our unaffected subjects. Furthermore, the dysbiosis index decreased over the course of the study, consistent with treatment and subsequent clinical improvement. When it was created, the dysbiosis index showed a strong correlation with clinical severity as measured by PCDAI, which we confirm in our study. We further share the novel finding that the dysbiosis index associates with the direct measure of inflammation: calprotectin. Because PCDAI does not show a similar association with higher calprotectin, the dysbiosis index may be more reflective of inflammatory status than the less direct disease activity measure.

Although our sample size is small, we showed that although the dysbiosis index was developed in patients with CD, patients with UC had significantly higher dysbiosis than did those with CD, along with increased calprotectin. Further, none of the responders in our study were UC patients. Additional studies in larger patient cohorts are needed to clarify any distinct features of the microbiome among IBD patients.

Our subjects were followed for an average of 8 months and included patients who both responded and did not respond to treatment. Although the dysbiosis index improved over time in patients, it did not reach levels seen in controls. This finding has important implications for pathogenesis: it suggests that with aggressive treatment of inflammation and symptoms (as was the case in our population) disease activity will improve, but the gut microbiome may remain perturbed. This finding is in line with a recent paper by Forbes et al., who found that there was no clear difference between microbiota of inflamed and non-inflamed mucosa in either CD or UC, suggesting that gut dysbiosis is the driver of inflammation rather than a result of it [37].

This pattern of persistent dysbiosis further emphasizes the need for prospective, longitudinal tracking with extensive follow-up: microbiome trends, microbiome resilience, and return to “healthy” composition may all be important to assess [38]. A larger study to investigate the impact of different treatments is also needed. Observations from such studies will open new therapeutic opportunities aimed at ameliorating dysbiosis in the hope of either preventing disease or limiting future complications.

At the individual genus level, several genera showed differences between groups in our GEE, random forest models, or both, with six bearing special mention: *Akkermansia, Coprococcus, Fusobacterium*, *Veillonella*, *Faecalibacterium*, and *Adlercreutzia*. In our sample, *Akkermansia* had a higher pretreatment abundance in non-responders compared to responders (Fig. 4b). The genome of *Akkermansia*, identified in our random forest analysis, contains mucinase genes [39] and is considered to be a mucin-degrading bacterium [40]. In gnotobiotic mice, *Akkermansia* increases inflammation in mice co-infected with *Salmonella typhimurium* [41]. We also found that *Coprococcus* (a genus identified in both GEE and random forest analyses) was diminished in cases compared to controls, and was further diminished in non-responders. In fact, agglutinating antibodies for *Coprococcus* were briefly considered as a biomarker for CD screening [42].

We have previously reported significantly higher abundance of *Fusobacterium* and *Veillonella* in the stool of treatment-naïve CD patients [19]. In our GEE analysis we again identified these two genera at increased abundance in cases, especially in non-responders to therapy. One recent study by Kelsen et al. identified significantly increased levels of these two taxa, among others, in the subgingival microbiome of patients with CD who were not taking antibiotics [43]. This prompts the hypothesis that oral cavity microbiota, also seen in the guts of IBD patients, may play a significant role in the pathogenesis and progression of IBD. Species of *Fusobacterium* are also associated with a wide variety of negative health outcomes, such as dental plaque, periodontal disease, Lemierre syndrome [44], head and neck infections [45], and especially colon cancer [46, 47].

*Faecalibacterium*, a genus of interest from our random forest analysis, includes the species *F. prausnitzii*. One particular strain of this species—A2-165—was recently found by Rossi et al. to have an important role in anti-inflammatory processes. This bacterium was particularly adept at eliciting high levels of IL-10 production, enhancing ovalbumin-specific T cell proliferation, and reducing interferon gamma-positive T cells. Treatment with A2-165 even attenuated inflammation in a murine model of chronic relapsing colitis [48]. Because *Faecalibacterium* abundance was found to be decreased in non-responders compared to responders, our study supports further investigation into the prognostic and therapeutic possibilities of this strain.

Another genus significant in both GEE and random forest analyses, *Adlercreutzia*, was found to be decreased in cases and further decreased in non-responders compared to responders. This genus was originally identified in human feces and found to play an important role in the metabolism of isoflavones to equol, a non-steroidal estrogen [49]. To our knowledge, the role of *Adlercreutzia* in IBD has not yet been explored; however, its appearance in the significant results of both our GEE and random forest analyses suggest it may be a future target of interest.

Genera from the families *Lachnospiraceae* and *Ruminococcaceae* appear several times in our GEE and random forest results. Though not included in the dysbiosis index, members of these families were found to be characteristic of tissue samples from Crohn’s disease in a recent study by Tyler et al. [50]. Four of the top 15 most important genera identified by our classifier belong to the family *Lachnospiraceae,* and all are reduced in non-responders compared to responders. Further research is needed into the possible contribution of members of this family to IBD pathophysiology.

Our study has several limitations. Some control subjects were related to affected subjects; however, the unrelated controls actually had significantly higher microbial dysbiosis than the related controls, suggesting shared environment did not overly inflate dysbiosis in the related study subjects. One factor that may have contributed to this trend is that some related controls were parents and were hence older than the affected subjects. Additionally, there was variation in the number of samples obtained from each patient. To correct for this variation, we weighted samples for each study subject according to the number of samples they contributed to the study. Our sample population had a smaller number of UC subjects than CD subjects; although patients with UC had higher measures of clinical activity, we combined these patients for predictive modeling, because IBD disease type did not explain a large proportion of the variance between microbiome samples among IBD cases.

These unique data provide the first glimpse into the long-term dynamics of the gut microbiome of subjects with and without IBD. The data show that the dysbiosis index captures alteration of the microbiome in IBD patients relative to controls, and associates with clinical and biochemical measures of disease activity. More importantly, the dysbiosis index did not decline to levels seen in unaffected individuals, even when patients were in remission. Distinct microbial signatures seen at the genus level among responders and non-responders may have clinical implications for therapeutics and risk stratification. The potential impact of this analysis is far-reaching, as it provides insight into how gut microbial dysbiosis changes with treatment and remission in patients with IBD. Our results also lay the groundwork for predicting patients’ ultimate response to therapy.

## Conclusions

### New findings

- Markers of inflammation and dysbiosis are increased in IBD; microbial dysbiosis improves over time but persists despite cessation of clinical disease activity and mucosal healing among responders.
- The dysbiosis index does associate with calprotectin, a measure of inflammation, but it does not distinguish treatment responders (those with mucosal healing) from non-responders. Other microbiome signatures do emerge at the genus level and warrant further investigation.

### Impact on clinical practice

- Treatment-naïve analysis of the microbiome could potentially be used to predict whether a subject will respond to treatment.
- Sustained and deep remission may require normalizing the gut dysbiosis.

## Abbreviations

AUC, area under the curve; CD, Crohn’s disease; GEE, generalized estimating equation; IBD, inflammatory bowel disease; OTU, operational taxonomic unit; PCDAI, Pediatric Crohn’s Disease Activity Index; PUCAI, Pediatric Ulcerative Colitis Activity Index; ROC, receiver operating characteristic; UC, ulcerative colitis

## Additional files

Additional file 1: Microbiome samples sequencing and analysis summary. Contains a table that summarizes total reads, reads passing QC, and reads successfully classified under closed and de novo OTU-picking approaches for each sample. Also provided are the dysbiosis and Shannon index values for samples under the aforementioned approaches along with median values after 10,000 rarefactions (subsamplings) of the closed-reference.biom table. (XLSX 46 kb) Additional file 2: Supplementary tables and figures. Contains supplementary tables and figures referenced in the paper. (DOCX 838 kb) Additional file 3: De novo and rarefy analysis. Contains a table that compares results from the generalized estimating equation (GEE) analysis performed in the paper (using closed-reference OTU picking) to de novo OTU picking and rarefied closed-reference approaches. (XLSX 10 kb)
