# Supplementary material for: Dysbiosis, inflammation, and response to treatment: a longitudinal study of pediatric subjects with newly diagnosed inflammatory bowel disease
Source: Genome Med. 2016 Jul 13;8:75. doi: 10.1186/s13073-016-0331-y (PMC4944441; doi:10.1186/s13073-016-0331-y)

**Supplementary information**

**Table S1:** summary of data available for all patients

|  |  | **Total number of observations** | | | **Overlap w/ microbiome** | |
| --- | --- | --- | --- | --- | --- | --- |
|  |  | **microbiome** | **calprotectin** | **PCDAI** | **calprotectin** | **PCDAI** |
| **Total** | **case** | 111 | 125 | 120 | 103 | 97 |
|  | **control** | 47 | 55 | 0 | 43 | 0 |
| **Median** | **case** | 6 | 6 | 7 | 5 | 6 |
|  | **control** | 5 | 6.5 | 0 | 5 | 0 |

**Table S2:** statistical comparison of related (reference group) and unrelated controls

calprotectin mean ± SD = 23.7 ± 60

Shannon index mean ± SD = 5.85 ± 0.61

dysbiosis index mean ± SD = -1.87 ± 0.58

| Difference at baseline: | | | |
| --- | --- | --- | --- |
| y ~ relation + time | | | |
|  |  |  | |
|  |  | X | |
|  |  | beta | p-value |
| Y | calprotectin | -20.9 | 0.5 |
|  | Shannon | -0.098 | 0.7 |
|  | dysbiosis | 0.32 | 0.1 |

| Average difference: | | | |
| --- | --- | --- | --- |
| y ~ relation | | | |
|  |  |  | |
|  |  | X | |
|  |  | beta | p-value |
| Y | calprotectin | -10.2 | 0.6 |
|  | Shannon | 0.033 | 0.9 |
|  | dysbiosis | 0.36 | 0.05 |

**Table S3:** statistical summary of differences between cases (stratified into CD and UC) and controls (reference group)

calprotectin mean ± SD = 240 ± 508

Shannon index mean ± SD = 5.24 ± 0.89

dysbiosis index mean ± SD = -1.28 ± 0.80

| Difference at baseline: | | | | | | | | | | | | | | |  |  |  |
| --- | --- | --- | --- | --- | --- | --- | --- | --- | --- | --- | --- | --- | --- | --- | --- | --- | --- |
| y ~ diagnosis(control/CD/UC) + time + diagnosis*time | | | | | | | | | | | | | | |  |  |  |
|  |  |  |  | |  |  |  | | | |  |  | | | |  |  |
|  |  |  | diagnosis | | |  | time | |  | diagnosis*time | | | |  |  |  |  |
|  |  |  | beta | | p-value |  | beta | p-value |  | beta | p-value | | |  |  |  |  |
| Y | calprotectin | CD | 313 | | 2E-04 |  | -0.069 | 0.4 |  | -1.03 | 0.02 | | |  |  |  |  |
|  |  | UC | 1330 | | 4E-11 |  |  |  |  | -4.15 | 3E-06 | | |  |  |  |  |
|  | Shannon | CD | -0.94 | | 1E-05 |  | -1.1E-03 | 0.3 |  | 1.8E-3 | 0.1 | | |  |  |  |  |
|  |  | UC | -1.31 | | 8E-05 |  |  |  |  | 6.3E-3 | 2E-03 | | |  |  |  |  |
|  | dysbiosis | CD | 0.86 | | 6E-08 |  | -7.1E-04 | 0.2 |  | -1.5E-3 | 0.03 | | |  |  |  |  |
|  |  | UC | 1.75 | | 4E-15 |  |  |  |  | -0.011 | 1E-13 | | |  |  |  |  |
|  |  |  |  | |  |  |  |  |  |  |  | | |  |  |  |  |
|  |  |  | (difference from controls) | | |  | (control change over time) | |  | (change over time compared to controls) | | | |  |  |  |  |
|  |  |  | |  | |  |  | | |  |  | |  | | | | |
| Average difference: | | | | | | | | | | | | | | |  |  |  |
| y ~ diagnosis(control/CD/UC) | | | | | | | | | | | | | | |  |  |  |
|  |  |  |  | |  |  |  | | | |  |  |  |  |  |  |  |
|  |  |  | diagnosis | | |  |  |  |  |  |  |  |  |  |  |  |  |
|  |  |  | beta | | p-value |  |  |  |  |  |  |  |  |  |  |  |  |
| Y | calprotectin | CD | 181 | | 2E-05 |  |  |  |  |  |  |  |  |  |  |  |  |
|  |  | UC | 1100 | | 4E-08 |  |  |  |  |  |  |  |  |  |  |  |  |
|  | Shannon | CD | -0.72 | | 7E-03 |  |  |  |  |  |  |  |  |  |  |  |  |
|  |  | UC | -0.98 | | 2E-03 |  |  |  |  |  |  |  |  |  |  |  |  |
|  | dysbiosis | CD | 0.67 | | 3E-07 |  |  |  |  |  |  |  |  |  |  |  |  |
|  |  | UC | 1.38 | | 3E-10 |  |  |  |  |  |  |  |  |  |  |  |  |
|  |  |  |  | |  |  |  |  |  |  |  |  |  |  |  |  |  |
|  |  |  | (difference from controls) | | |  |  |  |  |  |  |  |  |  |  |  |  |

**Table S4:** statistical summary of differences between UC and CD (reference group)

calprotectin mean ± SD = 335 ± 584

Shannon index mean ± SD = 4.99 ± 0.86

dysbiosis index mean ± SD = -1.03 ± 0.75

| Difference at baseline: | | | |
| --- | --- | --- | --- |
| Y ~ diagnosis(UC/CD) + time | | | |
|  |  |  |  |
|  |  | beta | p-value |
| Y | calprotectin | 829 | 2E-05 |
|  | Shannon | -0.18 | 0.5 |
|  | dysbiosis | 0.49 | 0.02 |

| Average difference: | | | |
| --- | --- | --- | --- |
| Y ~ diagnosis(UC/CD) | | | |
|  |  |  |  |
|  |  | beta | p-value |
| Y | calprotectin | 917 | 6E-06 |
|  | Shannon | -0.25 | 0.3 |
|  | dysbiosis | 0.70 | 7E-04 |

**Table S5:** statistical summary of the association between Shannon/dysbiosis and calprotectin/PCDAI, and between PCDAI and calprotectin

|  |  |  |  |  |  |  |  |
| --- | --- | --- | --- | --- | --- | --- | --- |
|  | **ALL CASES AND CONTROLS** |  |  |  |  |  |  |
|  |  |  |  |  |  |  |  |
|  |  | X | | | | | |
|  |  | Shannon | | dysbiosis | | | |
|  |  | beta | p-value | beta | p-value | | |
| Y | Calprotectin  mean ± SD = 266 ± 548 | -66.1 | 0.3 | 260 | 4E-04 | | |
|  |  | mean ± SD = 5.28 ± 0.86 | | mean ± SD = -1.3 ± 0.74 | | | |
|  |  |  |  |  |  |  | |
|  | **CASES ONLY** |  |  |  |  |  | |
|  |  |  |  |  |  |  |  |
|  |  | X | | | | |  |
|  |  | Shannon | | dysbiosis | | |  |
|  |  | beta | p-value | beta | p-value | |  |
| Y | Calprotectin  mean ± SD = 366 ± 626 | -13.3 | 0.9 | 286 | 3E-04 | |  |
|  |  | mean ± SD = 5.04 ± 0.84 | | mean ± SD = -1.06 ± 0.66 | | |  |
|  | PCDAI  mean ± SD = 13.1 ± 12.1 | -0.70 | 0.6 | 5.37 | 1E-04 | |  |
|  |  | mean ± SD = 4.97 ± 0.88 | | mean ± SD = -1.06 ± 0.75 | | |  |
|  | **CASES ONLY** |  |  |  |  |  |  |
|  |  | X | |  |  |  |  |
|  |  | PCDAI | |  |  |  |  |
|  |  | beta | p-value |  |  |  |  |
| Y | Calprotectin  mean ± SD = 241 ± 491 | 11.0 | 0.06 |  |  |  |  |
|  |  | mean ± SD = 12.4 ± 11.7 | |  |  |  |  |

**Table S6:** Significant OTUs in case/control and/or responder/nonresponder comparisons. OTUs highlighted in red are “increased” (numerator) components of the dysbiosis index, OTUs in blue are “decreased” (denominator) dysbiosis index components, and OTUs in grey are not represented in the dysbiosis index.

**Table S7:** The WEIGHTED random forest confusion table is presented below.

| **Confusion table** |  |  |
| --- | --- | --- |
|  | Nonresponder | Responder |
| Nonresponder | 11 | 1 |
| Responder | 3 | 2 |

**Table S8:** The random forest EQUAL SAMPLING confusion table is presented below.

| **Confusion table** |  |  |
| --- | --- | --- |
|  | Nonresponder | Responder |
| Nonresponder | 9 | 3 |
| Responder | 1 | 4 |

**Table S9:** The genera shown below had the 15 highest importance scores for classifying patients into treatment responders/non-responders as determined by WEIGHTED random forest.

**Table S10:** The genera shown below had the 15 highest importance scores for classifying patients into treatment responders/non-responders as determined by random forest with EQUAL SAMPLING. Genera in common with Table S8 are highlighted in red (*Dialister* was found previously in the top 15).

**Figure S1:** All time points for calprotectin (panel A), Shannon alpha diversity (panel B), and gut microbial dysbiosis (panel C) for unaffected controls (black circles), Crohn’s disease patients (CD, red circles), and ulcerative colitis patients (UC, blue circles) are shown. Overall, CD and UC patients have increased calprotectin, decreased alpha diversity, and increased gut microbial dysbiosis compared to controls.


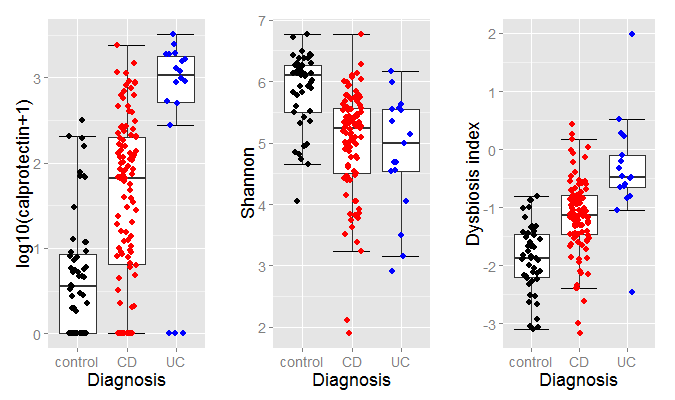


**A**

**B**

**C**

**Figure S2:** All control, responder, and non-responder calprotectin and microbiome time points further identified by individual.


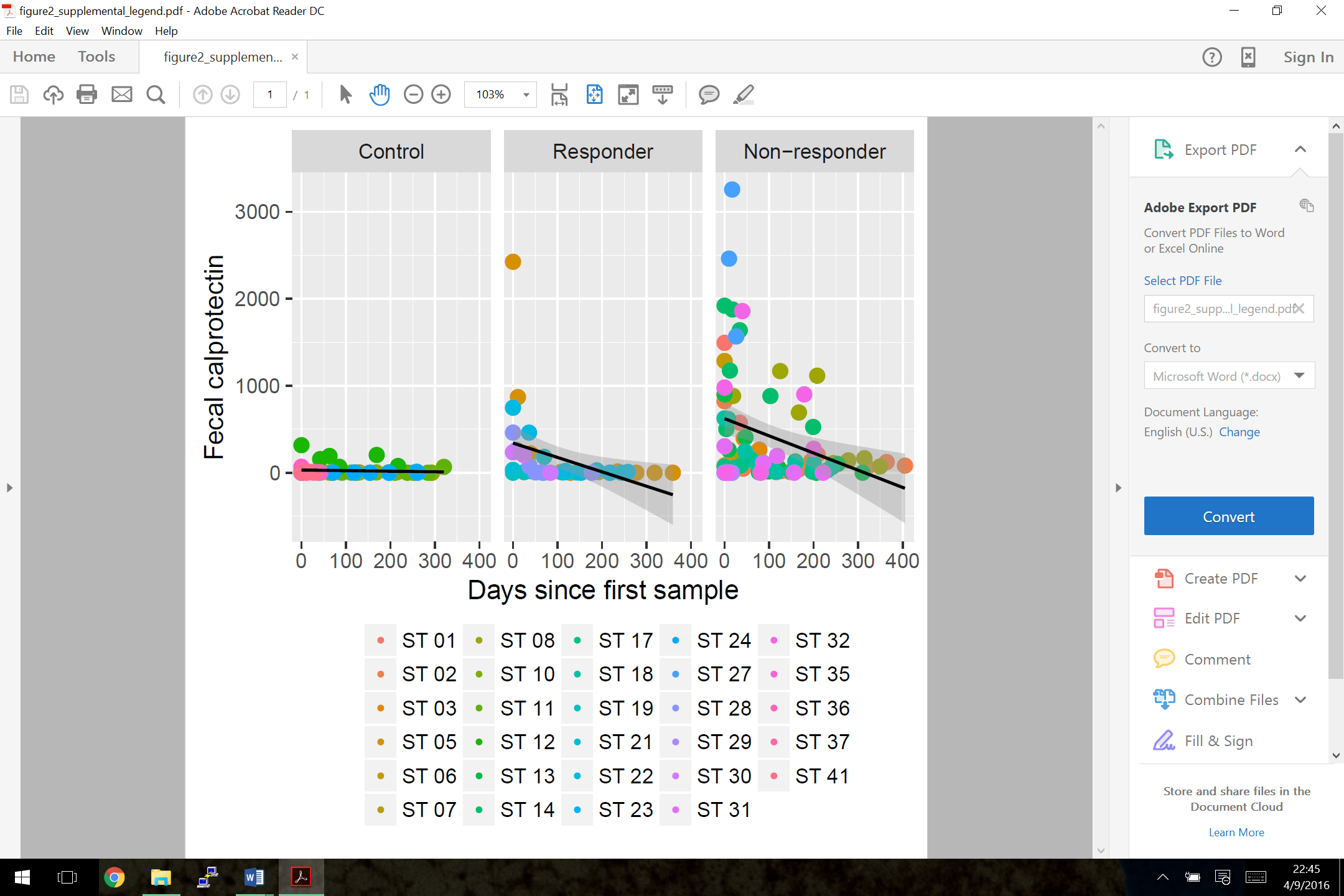

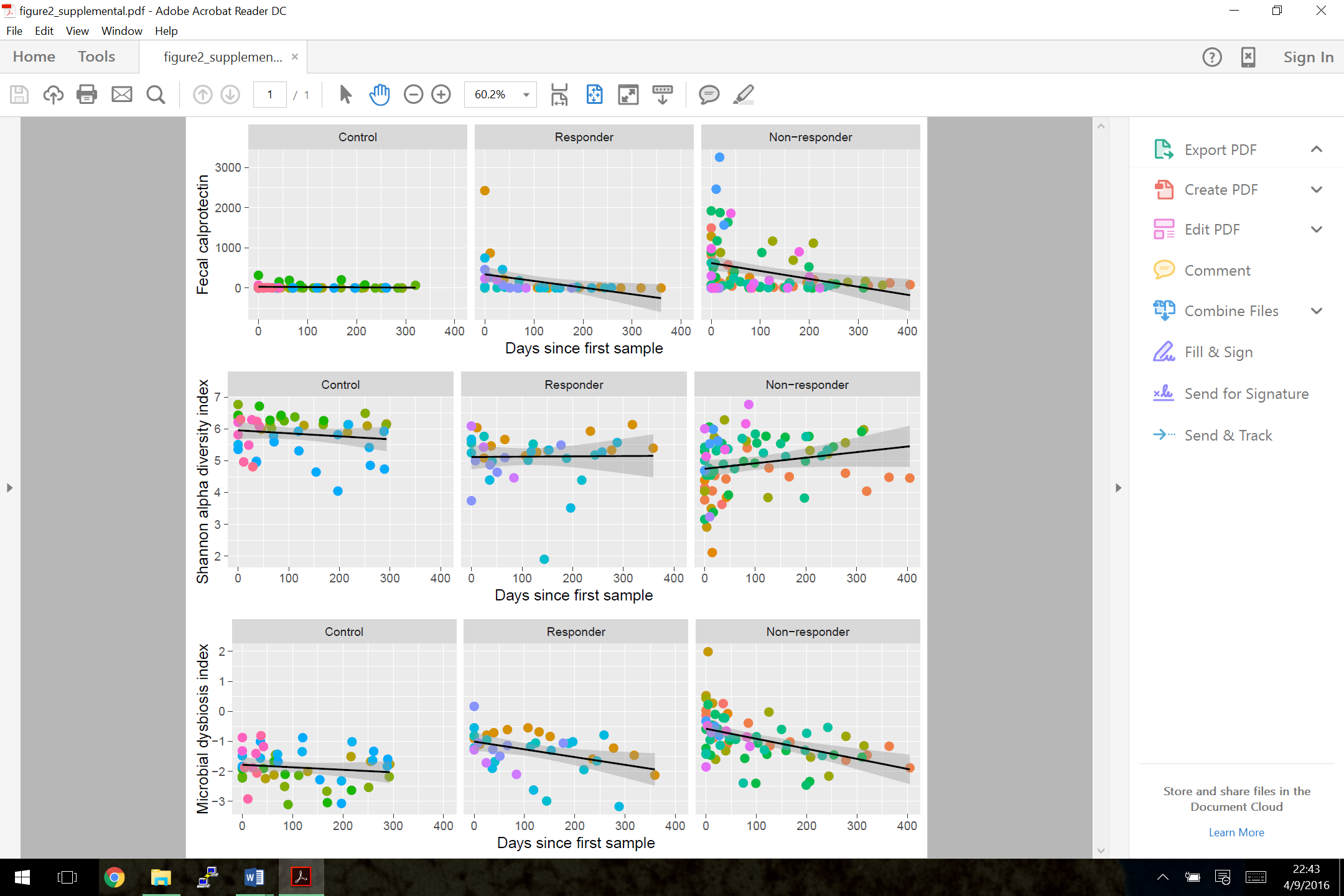


**Figure S3:** The relationships between relevant clinical measures for patients with IBD are shown. Regression lines, plotted in black, are adjusted for correlations within individuals. Dysbiosis and calprotectin are shown in panel A; panel B shows the relationship between dysbiosis index and PCDAI. Increased dysbiosis associates with increased calprotectin and higher PCDAI. Panel C shows the relationship between PCDAI and calprotectin. PCDAI does not significantly associate with increased calprotectin.


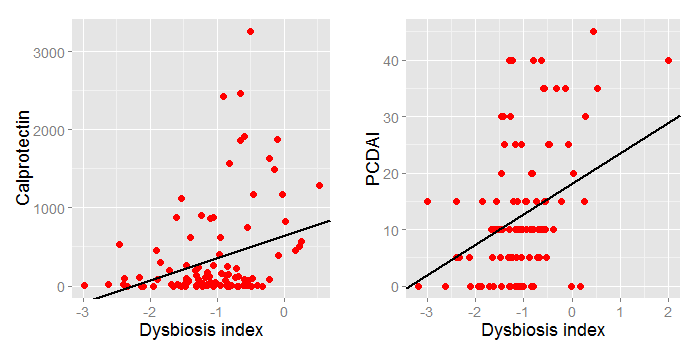


**B**

**A**


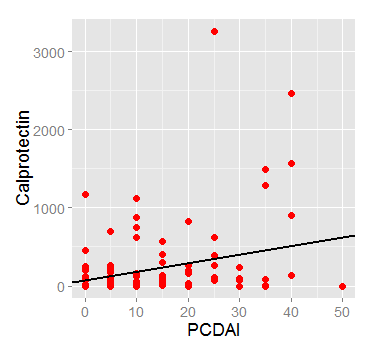


**C**

**Figure S4:** The precision (positive predictive value)/recall (sensitivity) curve for our weighted random forest model is shown below.


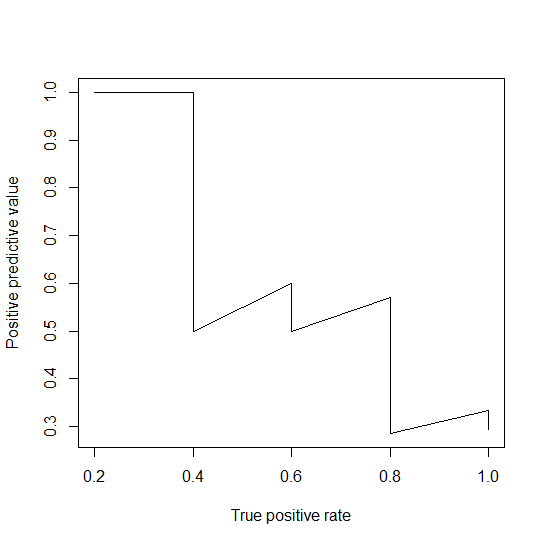


**Figure S5:** The receiver operating characteristic curve (ROC, panel A) and precision/recall curve (panel B) for our random forest analysis with equal sampling are shown below.


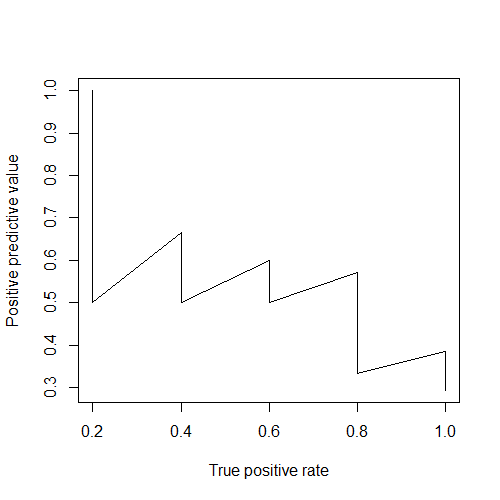

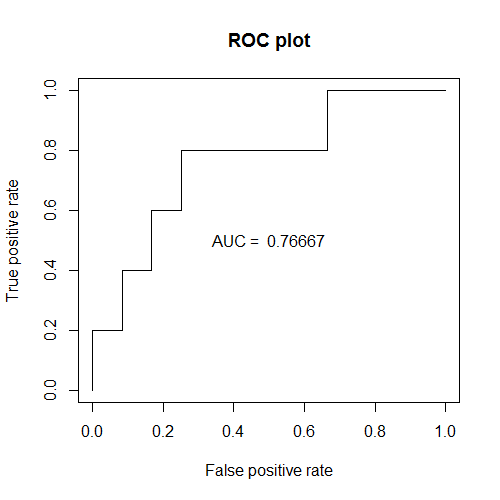


**B**

**A**

**Figure S6:** For those of the top 15 genera that were found above 1% average relative abundance, stacked bar charts are shown for each sample used in the random forest (categorized by response or non-response).


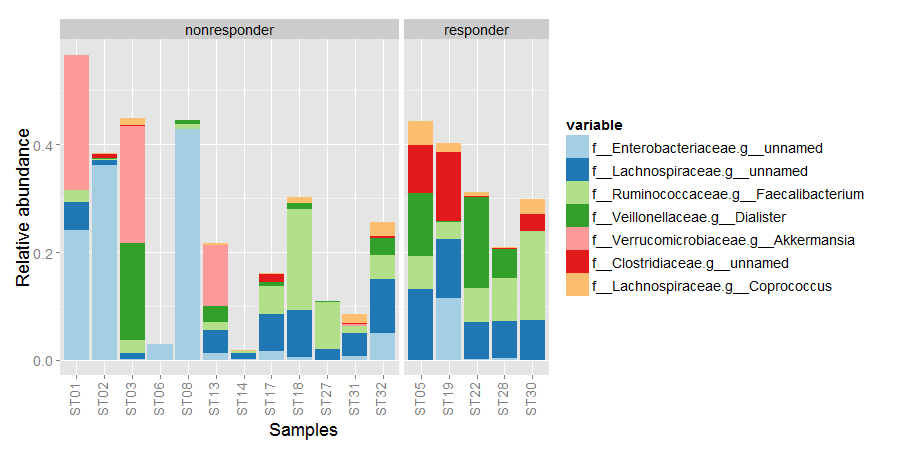

Supplement: Additional file 2: — Supplementary tables and figures. Contains supplementary tables and figures referenced in the paper. (DOCX 838 kb) [file 13073_2016_331_MOESM2_ESM.docx]
